# Contactless Ultrasonic Cavitation in Alloy Melts

**DOI:** 10.3390/ma12213610

**Published:** 2019-11-03

**Authors:** Koulis Pericleous, Valdis Bojarevics, Georgi Djambazov, Agnieszka Dybalska, William D. Griffiths, Catherine Tonry

**Affiliations:** 1 Centre for Numerical Modelling and Process Analysis, University of Greenwich, London SE10 9LS, UKC.Tonry@gre.ac.uk (C.T.); 2 School of Metallurgy and Materials, University of Birmingham, Birmingham B15 2TT, UK; A.Dybalska@bham.ac.uk (A.D.); W.D.Griffiths@bham.ac.uk (W.D.G.)

**Keywords:** ultrasonic treatment, contactless sonotrode, induction processing, grain refinement

## Abstract

A high frequency tuned electromagnetic induction coil is used to induce ultrasonic pressure waves leading to cavitation in alloy melts. This presents an alternative ‘*contactless*’ approach to conventional immersed probe techniques. The method can potentially offer the same benefits of traditional ultrasonic treatment (UST) such as degassing, microstructure refinement and dispersion of particles, but avoids melt contamination due to probe erosion prevalent in immersed sonotrodes, and it can be used on higher temperature and reactive alloys. An added benefit is that the induction stirring produced by the coil, enables a larger melt treatment volume. Model simulations of the process are conducted using purpose-built software, coupling flow, heat transfer, sound and electromagnetic fields. Modelling results are compared against experiments carried out in a prototype installation. Results indicate strong melt stirring and evidence of cavitation accompanying acoustic resonance. Up to 63% of grain refinement was obtained in commercial purity (CP-Al) aluminium and a further 46% in CP-Al with added Al–5Ti–1B grain refiner.

## 1. Introduction

Ultrasonic treatment (UST) of molten metals prior to solidification has been shown to improve their mechanical properties refining microstructure, degassing and dispersing strengthening particles [1]. The standard process involves the use of an immersed sonotrode probe vibrating at ultrasonic frequency (~20 kHz). Intense pressure waves are generated, which trigger dissolved gas cavitation that in turn assists nucleation [2], causes the break-up of intermetallics and evolving dendrites [3] and disperses clusters of particles. The process is, so far, mainly applied in low temperature melts (e.g., Al, Mg) but even then, there are problems preventing its widespread use by industry. The probe tip is dissolved at varying rates leading to melt contamination. The amount of liquid metal treated is concentrated in a small volume surrounding the probe, which necessitates additional mechanical stirring in order to spread the effect to a larger melt volume. Multiple sonotrodes often need to be used, making scalability complex and expensive [1].

To avoid these problems we devised the novel contactless electromagnetic (EM) UST process presented here. The new process eliminates the risk of melt contamination and the associated cost of frequent probe replacement, opening the potential benefits of UST to high temperature (e.g., Ni, Fe, Cu, ODS steel) or reactive (e.g., Ti, Zr) alloys. In contrast to the immersed sonotrode technique, where the kinetic energy of the vibrating horn is directly interacting with the liquid, the contactless EM device relies on acoustic resonance to reach pressure amplitudes leading to cavitation [4]. Furthermore, the Lorentz force, due to the induced current, leads to strong stirring, promoting multiple passages of the melt through active ‘*Blake threshold*’ pressure zones. Numerical simulations [4] indicate that scaling up to larger volumes can be achieved by simply introducing a larger coil and adjusting the current or frequency to match the acoustic resonance characteristics of the melt volume and surrounding container. Studying earlier publications [5,6], the idea of applying static or AC magnetic fields for the contactless ultrasonic treatment of liquid metals is not new, but the present implementation remains unique both in concept and in the ease with which it can be implemented in industry.

The remainder of the paper introduces the device known as the ‘Top Coil’ contactless sonotrode, summarises the mathematical methods used to model its function, followed by the experimental procedure together with sample results and discussion. Concluding remarks and references follow.

## 2. The Contactless Sonotrode

**Figure 1:**
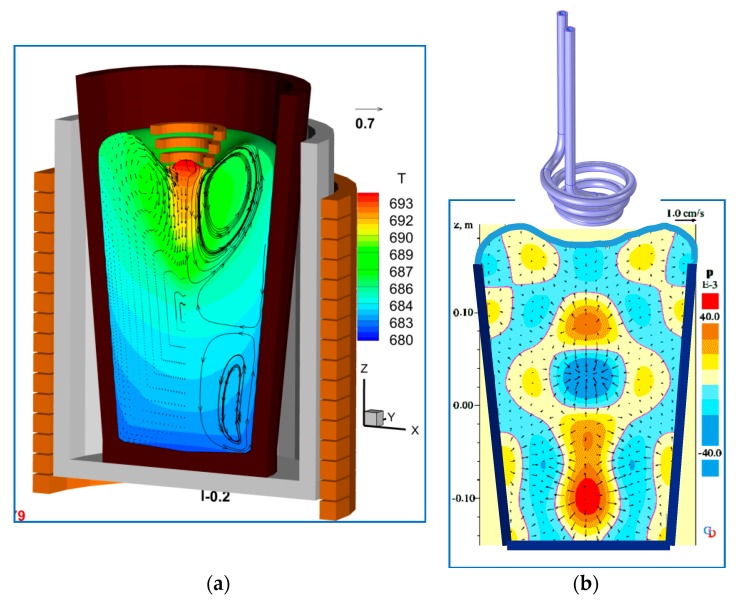
Simulations demonstrate the sonotrode concept [7]: (**a**) velocity and temperature <680–693 °C>, (**b**) instantaneous sound field < ± 40 kPa > with coil operating at 1000 A, 10 kHz.

The patented [7] ‘*Top Coil*’ sonotrode consists of a conical induction coil that can be lowered into the crucible containing the molten alloy, as shown schematically in Figure 1. The coil is water-cooled, with a current through it of sufficient magnitude to create a gap by EM repulsion between the liquid metal and the coil surface (typically ~1700 kA at ~9.5 kHz has been used for aluminium). A protective ceramic coating is employed as an additional safety feature to eliminate the risk of spark erosion.

Time-dependent simulations using a purpose-built spectral collocation code [8,9] coupling magnetic fields, turbulent flow and heat transfer in a dynamically varying fluid volume were used to design this system [4,7]. Details of the mathematical model used are given in Section 3 below. A typical simulation result in Figure 1, shows the dual effect of the Lorentz force arising from the interaction between the coil current and the opposing current induced in the melt: (i) In Figure 1a, the time-averaged component of the force repels the free surface and generates strong bulk stirring, (ii) the time-dependent component, acting at twice the supply frequency (see Equation (10)) vibrates the melt generating sound waves. The generation of sound waves is important in the process, since at the frequency needed for ultrasonic operation (~20 kHz), the applied EM force is concentrated in a thin ‘skin layer’ of fluid on the free surface (~7 mm in Al). Its effect can only be transmitted to the bulk through sound waves as pressure fluctuations, as shown in Figure 1b. In this case, the sound field was obtained by solving the compressible Euler momentum equations using a 4th order accurate finite difference scheme [10]. The amplitude of pressure fluctuations determines whether gas bubbles will emerge out of the solution, oscillate and under certain conditions cavitate. To reach the necessary pressure threshold for cavitation, the frequency of the coil needs to be tuned to produce resonance in the treatment vessel, accounting for the vessel geometry, free surface shape, temperature and crucible sound absorption characteristics. The use of resonance to enhance pressure amplitudes reduces the need for a very high current in the coil, as is found to be necessary in other proposed EM vibration techniques, for example in [5,6]. This latter fact makes the process energy efficient, especially where industrial scale operations are to be considered. The sound field simulations are needed to guide the frequency selection within the bounds of the power supply capacity.

In contrast to the immersed sonotrode technique where the cavitation energy is concentrated in a conical region surrounding the probe, in the proposed method, most cavitation activity is expected to lie in resonant nodes deep in the melt, were induced flow stirring will ensure that gas bubbles can have multiple passes through, improving cavitation efficiency. 

## 3. Mathematical Basis

It can be seen from the process description that this is a multi-physics application encompassing a range of traditional engineering fields. Due to space limitations the essential features of the models used are given here in summary with more detailed mathematical formulations given in the accompanying references. 

The set of equations representing fluid dynamics and heat transfer are solved using a spectral collocation scheme [8] on a dynamically varying solution grid [9] covering the liquid metal volume. The soundfield calculation domain includes the crucible and surrounding ambient region computed in the time domain, using a 4th order staggered variable scheme on a regular Cartesian grid [10]. Cavitation alters the speed of sound locally as the appearance of gas alters the medium compressibility. This aspect of the problem is handled using an extension of the Rayleigh–Plesset equations as suggested by Caflish [11] and implemented in [12,13]. 

### 3.1. Turbulent Fluid Flow and Heat Transfer

Characterised by the momentum and mass continuity equations, given by:

(1) $$\partial_{t}v+(v.\nabla )v=-\rho^{-1}\nabla p+\nabla \cdot (\nu_{e}(\nabla v+\nabla v^{T}))+\rho^{-1}j\times B+g$$

(2) ∇⋅v=0 

where, **v** is the velocity vector, *p* the pressure, *ρ* the density, *ν*_e_ the effective viscosity (sum of laminar and turbulent contributions), **j** the current density, **B** the magnetic field density, and **g** the gravity constant. The j × B term in Equation (1) represents the volumetric Lorentz force acting on the fluid.

(3) $$C_{p}^{}(\partial_{t}T+v\cdot \nabla T)=\nabla \cdot (C_{p}\alpha_{e}\nabla T)+\rho^{-1}|J{|}^{2}/\sigma$$

where, *T* is the temperature, *C_p_* the specific heat, *α_e_* the effective thermal diffusivity and *σ* the electrical resistivity of the liquid. The last term in Equation (3) represents the Joule heating generated by the induced current in the metal.

Turbulence is modelled by the k-*ω* Turbulence Model [14] (including magnetic field interaction):

(4) $$\partial_{t}k+v\cdot \nabla k=\;\nabla \cdot [(\nu +\sigma_{k}^{}\nu_{T})\nabla k]+G-\beta^{*}\omega \;k-\frac{2\alpha_{m}k}{\rho /(\sigma B^{2})}\;$$

(5) $$\partial_{t}\omega +v\cdot \nabla \omega =\;\nabla \cdot [(\nu +\sigma_{\omega }^{}\nu_{T})\nabla \omega ]+\alpha \frac{\omega }{k}G-\beta^{}\omega^{2}-\frac{\alpha_{m}\omega }{\rho /(\sigma B^{2})}$$

where, *k* is the kinetic energy of turbulence and *ω* its rate of dissipation. Note, standard nomenclature and model constants are used as in Wilcox [14].

### 3.2. Magnetic Induction

The AC magnetic field of angular frequency w due to the coil, ***B***, and the induced current, ***J***, can be divided into real and imaginary components.

(6) $$B\;=\;B_{R}\cos\omega t\;+\;B_{I}\sin\omega t\;$$

(7) $$J\;=\;J_{R}\cos\omega t\;+\;J_{I}\sin\omega t\;$$

where,

(8) $$J_{R}\;=\;\sigma \frac{\omega }{2}\delta \left(B_{R}\;+\;B_{I}\right);\;J_{I}\;=\;\sigma \frac{\omega }{2}\delta \left(-B_{R}+B_{I}\right)$$

The skin depth indicating current penetration into the fluid is given by

(9) $$\delta \;=\;\surd \;\left(\frac{2}{\mu \omega \sigma }\right)$$

where the magnetic permeability

μ(=μ0)=4π×10−7H/m

The Lorentz force is given by:

(10) $$F\;=\;J\times B\;=\;\bar{F}+\tilde{F}\;\bar{F}\;=\;\frac{1}{2}\left(J_{R}B_{R}+J_{I}B_{I}\right)\;=\;\frac{1}{2\mu \delta }B_{o}^{2}e^{-2\frac{x}{\delta }}\;\tilde{F}\;=\;\frac{1}{2\mu \delta }B_{o}^{2}e^{-2\frac{x}{\delta }}\sqrt{2}\cos(2\omega t-2\frac{x}{\delta }+\frac{\pi }{4})$$

As shown in Equation (10), the Lorentz force ***F*** being the cross product of magnetic field and current can be divided into mean and time-dependent (sinusoidal) components. The mean value is responsible for bulk stirring, while the sinusoidal part is the source of vibration. It is important to note (a) that the vibration frequency is double that of the supply current, (b) that the force decays within the skin-depth distance *δ* from the liquid free surface, hence the importance of resonance for achieving the required pressure amplitude for cavitation in the bulk volume.

**Figure 2:**
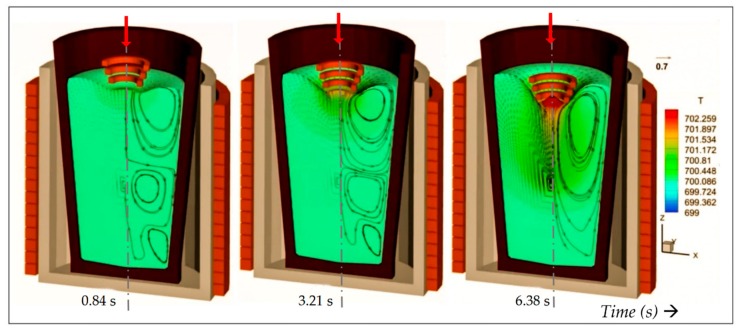
Predicted induced stirring and Joule heating in a typical aluminium crucible interacting with a descending conical coil. The main furnace coil operating at 2.4 kHz is switched of when the coil is in the lowest position, to maintain a maximum temperature of ~700 °C. Indicated temperature contour range < 699–702 °C. Maximum induced velocity is ~0.7 m/s.

Once the charge has melted with the aid of the main furnace coil surrounding the crucible, the top coil is moved axially down towards the free surface, in order to increase the EM coupling. The model simulations in Figure 2 show the process at three different time steps as the coil gradually deflects the free surface, together with the associated flow field and temperature distribution. The main furnace coil surrounds the crucible and contributes to stirring leading to the toroidal vortex pair appearing in the first two images. The top coil contributes to the melt temperature due to Joule heating, therefore to maintain the temperature at the optimum value for cavitation (in the case of aluminium around 700 °C), at some point in the process the furnace coil is turned off. In Figure 2, the stirring pattern is seen to change when this happens, as in the third figure on the right, where a single vortex dominates leading to deep recirculation in the crucible. Table 1 contains a working set of material properties used in the simulations.

### 3.3. Soundfield Computation

The mean Lorentz force component $\bar{F}$ is responsible for bulk stirring. The time-dependent component $\tilde{F},$ is the source of sound waves, computed by solving the Euler form of the momentum equations, generating a perturbation velocity field $\tilde{v}$:

(11) $$\frac{\partial p}{\partial t}+\rho c^{2}\frac{\partial \tilde{v}}{\partial x}=S;\;\rho \frac{\partial \tilde{v}}{\partial t}+\frac{\partial p}{\partial x}=\tilde{F}$$

A staggered scheme (in space and time) is used to solve Equation (11), with details are given in Djambazov et al. [10]. The source *S* represents pressure contributions due to cavitating bubbles [13]. The solution domain for sound extends beyond the melt to include the crucible and surrounding structures, thereby taking into account transmission and reflection of sound through the crucible walls. This means the acoustic impedance of all materials present needs to be considered. Constant pressure is assumed at the liquid free surface and a sound-hard boundary (zero flux) is applied at the coil surface. Details of the approach are given in [15]. 

A characteristic of the new process is the appearance of pressure nodes/antinodes deep inside the melt volume marking likely cavitation regions (e.g., see Figure 1b). This contrasts with the immersed sonotrode case, where cavitation activity is restricted to an area surrounding the vibrating probe, leading to shielding effects that limit process efficiency. 

## 4. Experimental Methods

**Figure 3:**
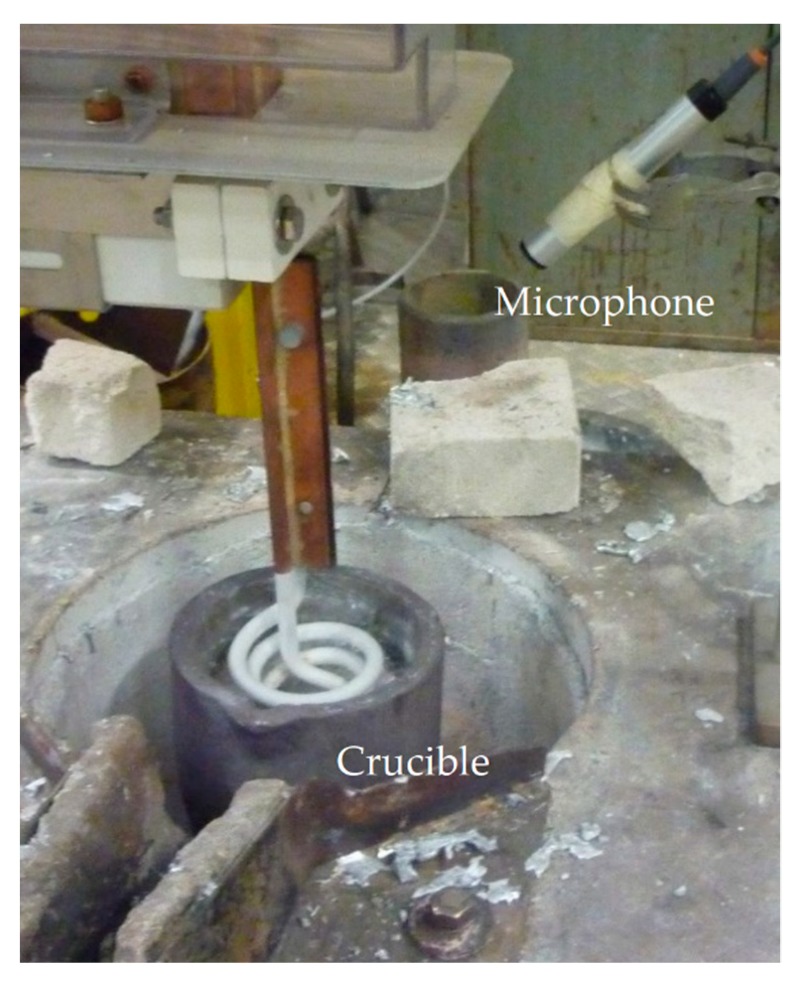
The ‘top-coil’ arrangement, showing the location of the high frequency microphone relative to the crucible.

Grain refinement experiments have been carried out using a cylindrical crucible investigating the top coil performance for CP-Al with and without added Al–5Ti–1B grain refiner. Numerical simulations were used in each case, to compute the optimum frequency for resonance, taking into account the melt volume, crucible geometry and acoustic properties of all materials present. Since the efficiency of the UST process depends on the extent of gas cavitation, a means of detecting cavitation activity in opaque liquids is necessary. In parallel research using immersed sonotrodes, we employed a specially commissioned cavitometer that operates through a long tungsten rod (the probe), providing thermal protection to the piezo sensing elements placed well outside the hot area, and with a bandwidth capable of capturing broadband acoustic emissions associated with cavitation activity [16]. Due to inductive pickup, the cavitometer could not be used with the top coil, relying instead on an externally mounted digital high frequency microphone, Ultramic®200K, to record sound emitted from the crucible and thereby detect cavitation activity. The experimental setup is shown in Figure 3. For reference, Table 2, gives the composition of alloys tested in various experiments.

### 4.1. Flowfield Validation

**Figure 4:**
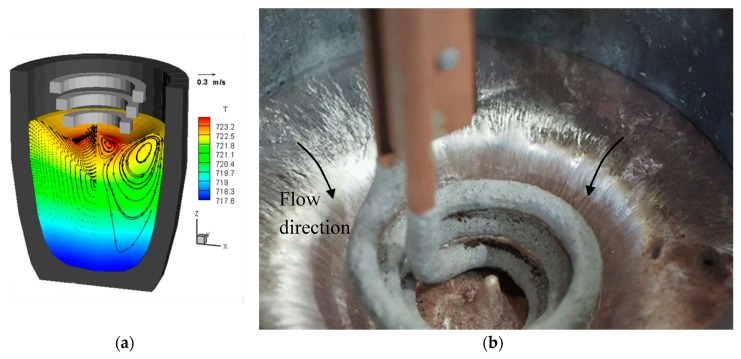
(**a**) Computed flow and heat transfer in a small 125 mm experimental crucible, containing A357 aluminium alloy used for particle tracking studies, (**b**) experiment showing the free surface of the melt; radial striations on the oxide layer indicate the flow direction and the conical elevation coinciding with the coil axis. Temperature contour range <717–723 °C>, maximum velocity 0.3 m/s.

Figure 4a shows the computed velocity and temperature field in a small conical 125 mm crucible containing A357 alloy used in experiments to follow the tracks of radioactive particles, using the PEPT technique [17,18] and Figure 4b shows, in a typical experimental result, the aluminium surface with the coil immersed in it. As predicted by the model, the free surface of the melt is depressed by the Lorentz force, preventing contact with the coil. Along the axis of the coil, the EM force vanishes, leading to the conical elevation of the surface, shown in both simulation and experiment. Strong radial motion was observed just below the thin layer of oxide (Figure 4b) in all experiments. This gave qualitative support to model flow predictions, of a dominant toroidal vortex pushing the liquid down close to the axis, and then returning near the periphery.

**Figure 5:**
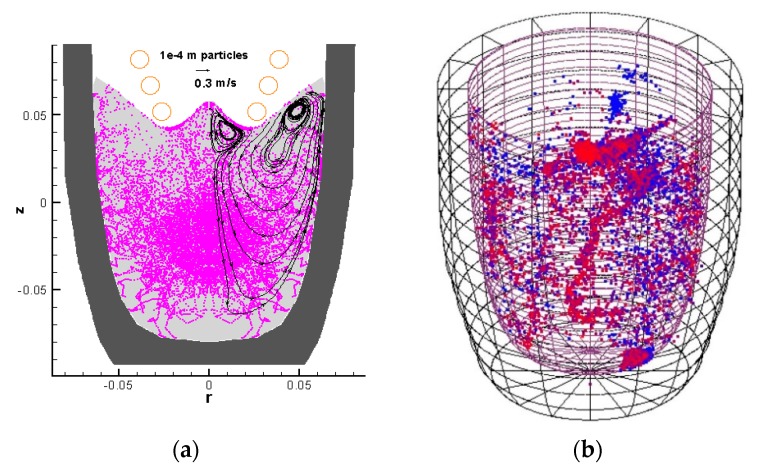
PEPT experiment, 125 mm crucible: (**a**) Simulation result showing dispersion of 100 μm particles due to induction stirring, (**b**) experimental 200 μm radioactive particle traces obtained using the PEPT technique.

The extent of stirring and therefore the ability of the top coil to disperse particles in the melt is clearly demonstrated in Figure 5. The numerical result shows 100 μm particle tracks obtained using Lagrangian tracking, accounting for the effects of turbulence and electromagnetic Kolin-Leenov forces [19]. In the simulation, particles seeded near the geometrical centre of the crucible are rapidly dispersed throughout the melt. The experimental result shows a similar dispersion pattern, obtained by tracking 200 μm radioactive particles using the Positron Emission Particle Tracking (PEPT) technique. From the processing point of view, this rigorous mixing is significant, since following initial cavitation, bubble or oxide fragments have the opportunity to re-enter the cavitation zone multiple times improving volumetric nucleation efficiency.

### 4.2. Grain Refinement

**Figure 6:**
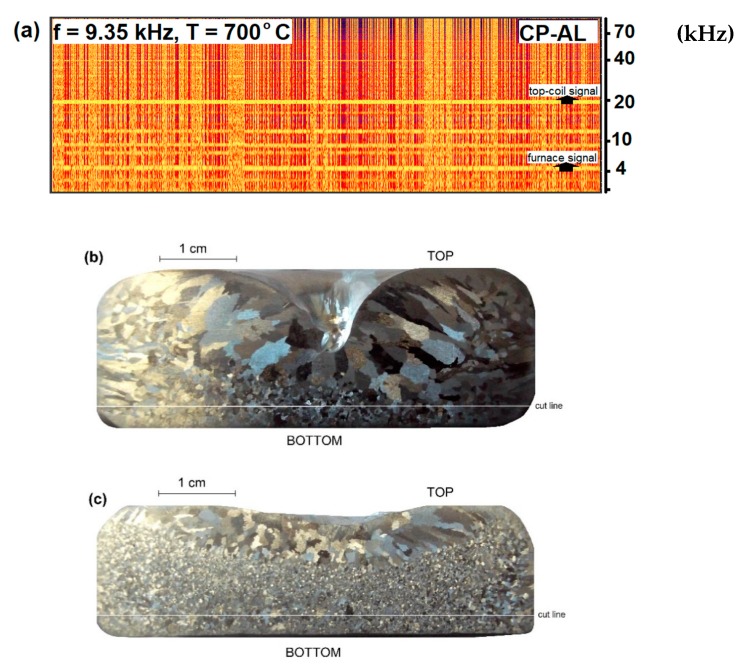
Grain refinement observed in CP-Al. (**a**) Recorded broadband noise during processing, (**b**) unprocessed sample, (**c**) sample processed by the contactless sonotrode at a frequency of 9.35 kHz at 700 °C. The cut line indicates the plane used for grain size measurements.

To evaluate the grain refining potential of the process, experiments were conducted in a cylindrical clay-graphite crucible with internal and external diameters of 135 and 170 mm respectively, and depth 280 mm. For each experiment the crucible was filled with about 8.5 kg metal, either commercial purity aluminium (CP-Al), or CP-Al with the addition of 0.2 wt.% Al–5Ti–1B grain refiner (100 ppm Ti, 20 ppm B). The top coil was positioned centrally above the liquid metal surface and during processing the ambient ultrasonic noise emitted around the crucible was recorded using the Ultramic®200K digital ultrasonic microphone. Recorded sound was observed in the form of a FFT (Fast Fourier transform) sound spectrum extracted in real-time during experiments using MatLab® software (R2014a). As mentioned earlier, detection of cavitation in an opaque medium is a non-trivial problem; one indicator of cavitation was the presence of broadband noise emitted by collapsing bubbles [20,21]. This was seen (Figure 6a) in the form of light-coloured vertical lines on spectrograms recorded over a period of 1–2 min. The lines appear normal to the continuous horizontal lines denoting the top-coil frequency signal, observed at around 20 kHz, and the induction furnace signal, observed at around 5 kHz. The number and density of vertical lines was considered to be a good indication of cavitation activity [22].

The conditions generating the noise, (coil frequency and melt temperature), were then maintained for a further 5 min to produce samples for grain structure analysis. The intensity of cavitation during the process should be reflected in the grain structure of the samples, which were taken using the KBI ring test [23]. In this test, liquid metal is poured into a steel ring with an outside diameter of 75 mm, inside diameter 50 mm and height 25 mm, placed on an insulating silica brick. The cast sample is then subject to three simultaneous modes of cooling: through the air, the steel mould, and the silica brick. As the tuned resonant frequencies were shown by the simulations to be dependent on melt volume [22], the KBI ring test was the most useful for the contactless sonotrode experiments, as it only requires small samples, of about 50 g of Al. For grain size characterization, the base of the cylindrical samples was removed to about 3 mm above the bottom and ground, polished and etched with either Poultons’ or Kellers’ solution. The average grain size was then determined using the mean linear intercept method.

**Figure 7:**
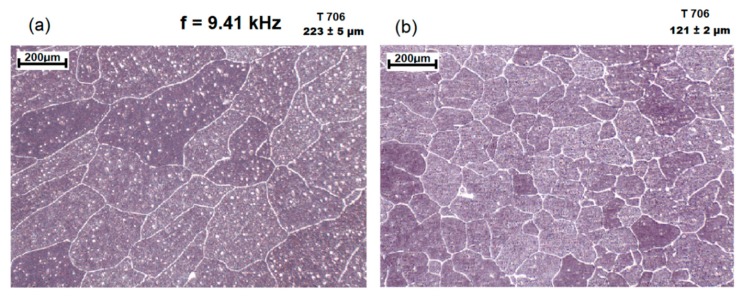
Grain size reduction following processing of sample containing commercial grain refiner: (**a**) the sample with Al–5Ti–1B addition, (**b**) alloy additionally processed by the contactless sonotrode with frequency 9.41 kHz for about 5 mins (Both samples were cast at 706 °C).

Figure 6 and Figure 7 show the recorded spectrograms and post mortem grain structures of samples obtained following ultrasound treatment.

Figure 6b,c show the grain refinement achieved with CP-Al at 700 °C in a 150 mm diameter cylindrical crucible with 1700 A, 9.35 kHz current through the coil, corresponding to the spectrogram in Figure 6a. It was found that one of the factors that had to be controlled during ultrasound processing was the melt temperature, which must be kept low to promote cavitation. In these experiments it was maintained at 40 °C above the melting point, which for pure Al was 700 °C, the minimum value at which it was possible to pour the liquid metal [24] in a casting. Since the cavitation process starts with the formation of bubbles, the solubility of hydrogen gas in the liquid Al is an important factor in the process. Solubility decreases with temperature, so at lower temperatures, the existence of stable bubbles is more probable [1,25]. In the case of CP-Al (Figure 6), the grain size reduction was about 63%, (a reduction from 256 ± 12 to 95 ± 1 µm). This level of performance is consistent with previous findings where 70% grain size reduction was obtained [22], measured, in that case, by using the Aluminium Association Test Procedure-1 (TP1).

There are several reasons given in the literature for the observed reduction in grain size due to cavitation. Cavitation is believed to induce heterogeneous nucleation by (i) forced wetting of non-wetted particles present in the melt, resulting in an increased number of nucleation sites [26,27], (ii) local undercooling due to pressure changes when bubbles collapse [28] or (iii) undercooling of the melt at the bubble surface when the bubble rapidly expands [29]. In the case of CP-Al, the number of non-wetted particles should be smaller than in the case of alloys with grain refiner addition, a fact that makes grain refinement more difficult. 

The addition of grain refiner increased the number of active nuclei and therefore all three mechanisms of cavitation-induced heterogeneous nucleation can take place. Figure 7 shows the grain size reduction achieved for CP-Al with a grain refiner. Grain sizes decreased from 223 ± 5 to 121 ± 2 µm.

The experiments used a 0.2% Al–5Ti–1B ternary master alloy, which is commonly adopted as a grain refiner for most aluminium alloys [30,31]. Using the same alloy section as in Figure 6, grain sizes of the base alloy are shown in Figure 7a and the reduction caused by ultrasound shown in Figure 7b.

### 4.3. Correlation Between Grain Refinement and Frequency Spectrum

**Figure 8:**
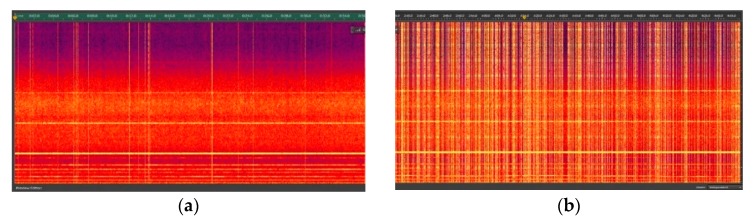
Contrasting nature of cavitation in two cases with very similar generator frequency, in (**a**) 9.32 kHz, in (**b**) 9.42 kHz obtained in a crucible with internal diameter 140 mm (remembering the vibration frequency in the coil will be doubled in the melt (10)).

The basic concept behind the contactless sonotrode relies on the initiation of gas cavitation activity using acoustic resonance as the main driver for grain refinement. Numerical simulations provide an indication of the likely resonant modes given the sound properties and geometry of the alloy and crucible materials [15,32]. However, since these properties can vary unpredictably (especially so in ceramic crucibles), the experiment traverses the space about the indicated central frequency value using the spectrogram as an indicator of the most potent value, judged by the frequency of broadband noise bursts. Examining the results obtained in the larger 140 mm diameter crucible with internal depth 300 mm, we see for example, with reference to Figure 8, that spectrogram (a) (coil frequency 9.32 kHz) shows no cavitation activity, whilst spectrogram (b) (coil frequency 9.42 kHz) shows a dense pattern of cavitation bursts. Also evident in all spectrograms is that the cavitation activity as shown by the vertical lines is intermittent. This may be due to local changes in sound velocity in the melt as clouds of bubbles appear and disappear, which would disrupt the resonant conditions.

**Figure 9:**
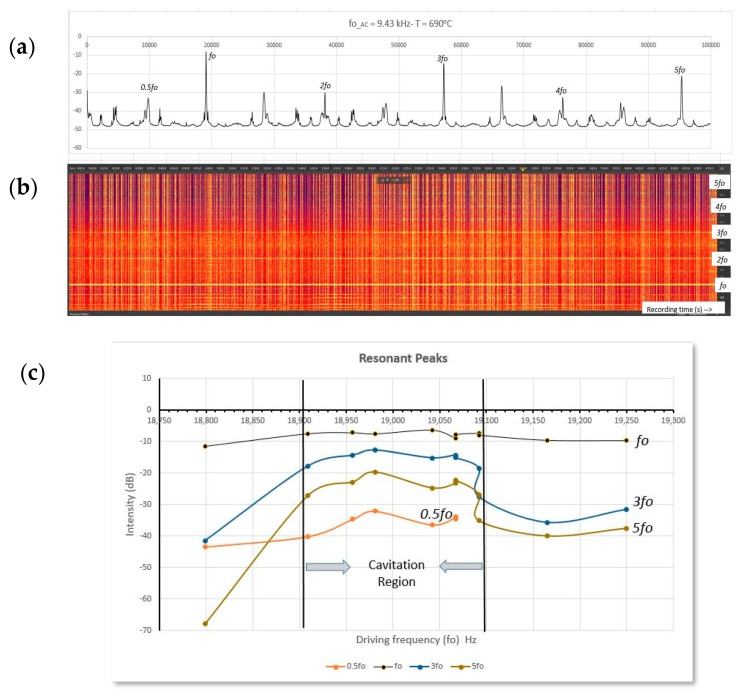
(**a**) FFT spectrum showing resonant peaks for 9.43 kHz (nominal) coil current, (**b**) corresponding spectrogram identifying horizontal lines as functions of driving frequency, (**c**) resonant peak intensity variation versus driving frequency (18,800–19,250 Hz), showing the cavitation region.

It is then interesting to examine the sound wave resonant nodes that are most likely to excite cavitation. Figure 9 scans the range between 9.32 and 9.56 kHz applied to the 140 mm diameter cylindrical crucible containing CP-Al. In Figure 9a a typical FFT for the fairly active 9.43 kHz experiment identifies strong sound peaks at the driving frequency *f*o and its 3rd and 5th harmonics, an indicator of axial (up and down) wave reflections. The radial waves identified by the even harmonics are much weaker. Figure 9b Shows the spectrogram for the 9.43 kHz case, identifying the various peaks as horizontal lines (i.e., persisting in time). Finally, in Figure 9c the peak amplitude for the various harmonics was plotted against the driving frequency. The cavitation region coincides with the bulge in the 3rd and 5th harmonics amplitude, between 18,900 Hz and 19,100 Hz.

## 5. Concluding Remarks

This paper shows a contactless electromagnetic processing technique that can generate ultrasonic waves in liquid metals in a crucible strong enough to produce cavitation. Originally developed as a theoretical concept, this technique was tested experimentally in the treatment of liquid aluminium alloys. The computational model which couples fluid flow, heat transfer, electromagnetics and soundfield simulations was validated in aspects that are important for the process by the experimental results. These confirm the predicted free surface depression by the coil, induction driven flow leading to strong mixing and the presence of cavitation. It was found that acoustic resonant conditions are necessary to produce pressure waves of sufficient strength for cavitation, which means geometrical details and material sound properties of the setup become important for successful implementation. Using this technique, it has been possible to produce grain-refined samples of both pure aluminium and aluminium with grain refiner added.

Although the experiments presented so far are conducted in aluminium, since the technique is contactless, it should be equally applicable to high temperature or reactive metals, such as steels, nickel alloys and titanium, were the immersed sonotrode technique cannot be used. This is a subject of continuing research.

## Figures and Tables

**Table 1:** Material properties of aluminium.

Material Property	Aluminium (700 °C)
Sound Speed c (m s^−1^)	4600
Density *ρ* (kg m^−3^)	2350
Dynamic Viscosity μ (mPa s)	1.3
Surface Tension *γ* (N m^−1^)	0.87
Thermal Conductivity *λ* (Wm^−1^K^−1^)	92
Electrical Conductivity *σ* (Sm^−1^)	3.8 × 10^7^
Specific Heat *C*_p_ (kJ kg^−1^ K^−1^)	1.18

**Table 2:** Composition of alloys tested.

Alloy	Si	Mg	Ti	Cu	Fe	Be	Mn	Zn	Balance Al
**A357**	6.5–7.5	0.55–0.6	0.1–0.2	0.0–0.2	0.1	0.002	0.1	0.0–0.1	90.8–93.0
**CP-Al**	0.3	0.03	0.0	0.03	0.4	0.0	0.03	0.07	99.5
